# Transcriptomic Analyses and Potential Therapeutic Targets of Pancreatic Cancer With Concomitant Diabetes

**DOI:** 10.3389/fonc.2020.563527

**Published:** 2020-11-04

**Authors:** Tingting Xu, Xiaoxuan Xu, Peng-Cheng Liu, Hui Mao, Shenghong Ju

**Affiliations:** ^1^ Jiangsu Key Laboratory of Molecular and Functional Imaging, Department of Radiology, Zhongda Hospital, Medical School of Southeast University, Nanjing, China; ^2^ The College of Life Science, Anhui Normal University, Wuhu, China; ^3^ Department of Radiology and Imaging Sciences, Emory University, Atlanta, GA, United States

**Keywords:** type 2 diabetes mellitus, pancreatic cancer, obesity, mouse model, transcriptomic analysis, matrix metalloproteinases

## Abstract

**Background:**

Type 2 diabetes mellitus (T2DM) known as non-insulin-dependent diabetes mellitus, which is increasingly acknowledged as being associated with an increased risk for a series of cancers. Pancreatic cancer is currently the fourth most common cause of cancer-related mortality, which has been proved to be worsened by internal diabetic condition. However, the underlying molecular mechanisms are less addressed. Furthermore, current knowledge revealed that therapeutic strategy by anti-diabetes for pancreatic cancer under diabetes condition have no satisfactory efficacy, and nor by chemotherapy in our study.

**Methods:**

To clarify these mysteries and widen our knowledge, both obesity-associated and non-obese-associated T2DM mouse models were generated by chemical induction with streptozotocin (STZ) and leptin receptor knockout (*db/db*) in mice. Then, the process of tumor progression was researched, and the gene expression profiling of pancreatic cancer in mice was performed using RNA-seq.

**Results:**

Our results showed that pancreatic cancer malignancy was increased with notable proliferation and metastatic potential in two diabetic mice model. Totally, 136 and 64 significantly differentially expressed genes (DEGs) were identified in STZ and *db*/*db* mice by transcriptomic analysis. The results also suggested that different carcinogenesis-related genes and potential molecular mechanisms contribute to the malignancy of pancreatic cancer in obesity-associated and non-obesity-associated T2DM. In obesity-associated *db*/*db* mice, the GO subcategories associated with most of the genes with downregulated expression are involved in the immune response. However, in non-obesity-associated STZ mice, in addition to the immune response category, the enriched subcategories also included angiogenesis and the extracellular matrix. While, two genes respectively encoding MMP-2 and MMP-9 were simultaneously abnormal up-regulated in pancreatic cancer tissue from diabetic mice of both STZ and *db*/*db*, that could act as potential therapeutic targets for significantly suppressing the malignant progression. Furthermore, an optimizing therapeutic strategy was further proposed that combining MMP-2/9 inhibitor with gemcitabine significantly enhanced anti-tumor effects on pancreatic cancer under diabetic condition, providing a theoretical basis for clinical applications.

**Conclusions:**

Generally, this study provides a comprehensive insight into diabetes as a risk factor for pancreatic cancer and has the potential to guide the development of enhanced treatment strategies.

## Introduction

Type 2 diabetes mellitus (T2DM) is known as non-insulin-dependent diabetes mellitus or adult-onset diabetes and is the most common form of diabetes, affecting approximately 90% to 95% of all patients diagnosed with diabetes (1). With the rapid increase in the incidence of diabetes worldwide, the prevalence of diabetes in combination with cancer is increasingly being observed (2, 3). Convincing evidence indicates that diabetes, particularly T2DM, is associated with an increased risk of the development and progression of several cancers, such as breast, liver, pancreas, colorectal, and bladder cancer (4, 5). A possible explanation for the link between cancer and diabetes is that these two disorders share many potential modifiable (e.g., weight change, diet, physical activity, tobacco smoking, and alcohol) and non-modifiable (e.g., sex, age, and race) risk factors (6, 7). As research has progressed, an increasing number of studies have focused on the possible biological links between diabetes and cancer, including several mechanisms such as hyperinsulinaemia (either endogenous or exogenous), hyperglycaemia, and chronic inflammation (8–11). In addition, another critical issue regarding the association between T2DM and cancer is whether drug use in patients with T2DM is causally associated with cancer. Clinically, medication-based diabetes treatments have been used, and a reduced risk of developing cancer has been reported in patients treated with several anti-diabetic drugs, such as metformin (12). However, anti-diabetes strategies may also not prevent or may even promote cancer development. For example, clinical trial evidence has shown that an increased malignancy rate is associated with the long-term use of exogenous insulin (13), and the postulated links between incretin-based therapies, such as glucagon-like peptide-1 receptor agonists and dipeptidyl peptidase-4 inhibitors, and pancreatic cancer have not been substantiated (14).

Pancreatic cancer is currently the fourth most common cause of cancer-related mortality (2, 15), and predictions indicate that it will be the second leading cause of cancer-related mortality within the next decade (3). Most patients are diagnosed at an advanced stage with distant metastasis and/or locally advanced unresectable tumors, and the 5-year survival rate is less than 7% (16). Diabetes mellitus has been identified as a risk factor for pancreatic cancer, causing increased morbidity and mortality and manifesting as an increased tumor size and reduced survival (17–20). In addition, some research has suggested that diabetes is both a cause and a consequence of pancreatic cancer (21), as pancreatic cancer itself induces diabetes (type 3c). The potential mechanisms of this process include the release of adrenomedullin, a potential mediator of beta cell dysfunction (22) and beta cell apoptosis induced by pancreatic stellate cells (23). Currently, anti-diabetes therapeutic strategies for pancreatic cancer patients with diabetes are not effective (4, 24–26), and there is a lack of effective therapeutic strategies.

Carcinogenesis is a complex process that involves multiple steps, including initiation, promotion and progression. A number of genes and proteins, for example, KRAS, CDKN2A, TP53, HER2, and HIF-1α, have been indicated to play important roles in those steps (27–30). One or more genes and proteins might be more abnormally expressed in pancreatic cancer with concomitant diabetes, resulting in the promotion of cancer progression. Thus, in this study, we attempted to identify potential genes and proteins that were abnormally expressed and then to target/suppress these genes and proteins to search for a more effective therapeutic strategy. To search for abnormally expressed genes, transcriptomic analyses were performed. In addition, being overweight or obese is considered the principal modifiable risk factor for diabetes (31); in 2010, 84.7% of adults aged ≥18 years with diagnosed diabetes in the United States were overweight or obese (32). Diabetes is an increasingly prevalent chronic condition in Asia despite low obesity rates and low body mass indexes (BMIs) (33, 34). Thus, to completely account for the various types of T2DM in humans, obese and nonobese mouse models of diabetes were established and studied.

## Materials and Methods

### Animal Models of Diabetic Pancreatic Cancer

All animal experiments were approved by the Institutional Animal Care and Use Committee of the Medical School of Southeast University. All mice were purchased from Kawensi Limited Company in Changzhou, China. Wild-type C57BL/6J mice (male, 8–10 weeks old) were used as nondiabetic controls (WT). Diabetic mouse models were generated either by chemical induction with streptozotocin (STZ) or through systemic mutations of leptin receptor (*db/db*). Since STZ-induced diabetes is not considered to be completely representative of T2DM in humans, especially obesity-associated T2DM, *db/db* (male, 8–10 weeks) and STZ mice were used to simulate the pathophysiology of obesity- and non-obesity-associated T2DM, respectively. Mice with the *db/db* genetic background develop hyperglycaemia as a consequence of obesity due to the deficiency in leptin receptor and beta cell failure and naturally exhibit many of the symptoms observed in obesity-associated T2DM patients. The STZ mouse model of diabetes was constructed as described by Chow et al. (35). The weights of the animals in the different model groups were measured. The weights of *db*/*db* mice were significantly higher than those of WT mice, whereas those of STZ mice were significantly lower than those of WT mice.

To verify that T2DM was successfully induced in these animals, glucose levels in blood collected from the tail vein were measured using a glucometer (Roche Diagnostics GmbH, Mannheim, Germany). The results showed that the glucose levels in WT mice were 6.88 ± 0.57 mmol/L (n=6), which were significantly lower than those in diabetic mice from the STZ (n=7, 23.67 ± 2.92 mmol/L; *t*=-5.212, *df*=11, *p*<0.001) and *db*/*db* (n=10, 20.65 ± 2.12 mmol/L; *t*=-4.893, *df*=14, *p*<0.001) groups.

Orthotopic pancreatic cancer models were generated in WT, STZ and *db*/*db* mice as previously described (36). Briefly, murine pancreatic cancer panc02 cells purchased from the Cell Bank of Shanghai Institute of Biological Science (Shanghai, China) were cultured in Dulbecco’s modified Eagle’s medium (DMEM) with 10% foetal bovine serum (FBS) and 1% penicillin and streptomycin. To monitor tumor development, migration, and therapeutic response *in vivo*, optical imaging reporter molecules were introduced into Panc02 cells with effective lentiviral infection procedures that were previously used in our laboratory (37). Briefly, with the FUGW-Luc2-eGFP construct generated using an hUbC-driven eGFP lentiviral plasmid and an FUGW vector, Panc02 cells were labelled with green fluorescent protein (GFP) and firefly luciferase dual reporters. Then, GFP-positive cells were sorted using a FACSCalibur instrument (BD Biosciences, USA). Panc02 cells were harvested and resuspended as single-cell suspensions in 100 μl and then were slowly injected into the distal femurs of WT mice. When subcutaneous tumors reached 8–10 mm in diameter, 1-mm3 tissue fragments were surgically resected from the subcutaneous tumor graft and were implanted into the pancreatic tail of WT STZ and *db*/*db* mice to obtain orthotopic pancreatic cancer models.

### Pancreatic Cancer Progression in Diabetic Mouse Models

Tumor growth was monitored *via* magnetic resonance imaging (MRI) using a 7.0 Tesla MR scanner (Bruker Pharma Scan; Ettlingen, Germany) with a transmit–receive quadrature volume coil. T2-weighted imaging (T2WI) using a fast spin-echo sequence with respiratory gating (echo time 36 ms) was performed on mice for 12 days after tumor implantation. The acquisition parameters included a field of view of 32×32 mm, a matrix of 256 × 256, and a total of 24 slices with a 1-mm slice thickness. Tumor length and width were manually measured in the largest cross-sectional area of the T2-weighted images to calculate the tumor volume according to the formula V=(width^2^×length)/2 (38).

At the end of the MRI experiment, mice were anaesthetized for *in vivo* bioluminescence imaging. Before imaging, D-luciferin (intraperitoneal (i.p.), 150 mg/kg; Promega, USA) was administered and allowed to react with the substrate for 10 min. An IVIS Spectrum imaging system (PerkinElmer, USA) was used to acquire bioluminescence images. Abdominal metastases were counted in the bioluminescence images.

### Therapeutic Schedule for Gemcitabine

A standard treatment for pancreatic cancer, gemcitabine (Sigma, USA), which is the first-choice chemotherapy agent for pancreatic cancer (39), was used to treat WT (n=10), STZ (n=7), and *db/db* (n=8) mice. Mice were administered gemcitabine (25 mg/kg, i.p.) every 3 days for 20 days. The tumor volume and metastases were evaluated using the methods described above. The control groups mice were administered an equal volume of vehicle (10% DMSO).

### Transcriptomic Analyses

For the transcriptomic experiment, tumor tissue (3 mm^3^) that was transplanted in WT, STZ and *db*/*db* mice for 12 days was collected and pooled in a plastic tube (1.5 ml), snap-frozen in liquid nitrogen, and transferred to a -80°C freezer for long-term storage. RNA from each sample group was extracted with TRIzol reagent (Invitrogen; USA), and each group included three replicates. The quality of the isolated RNA was assessed using a NanoDrop (Thermo Scientific NanoDrop 2000, USA), and the A260/280 values were all above 2.0. A total of 3 μg of total RNA from each sample was converted to cDNA using an NEBNext^®^ Ultra™ RNA Library Prep kit for Illumina^®^ (NEB, USA). In total, nine cDNA libraries were constructed and subsequently sequenced with an Illumina HiSeq 2000 platform by Beijing Biomarker Technologies Co., Ltd., resulting in raw reads. Clean reads were obtained by removing reads containing adapters, poly-N reads and low-quality reads from the raw data using FASTX-Toolkit (http://hannonlab.cshl.edu/fastx_toolkit/), and these clean reads were used for further analyses. Approximately 44.24 million clean reads were obtained from each sample. The Q30 was greater than 93.57% in each sample, showing that the sequences of each sample were of high quality.

The transcriptome was assembled using StringTie (40) based on the reads that mapped to the reference genome GRCm38; more than 93.18% of the reads were successfully mapped. In addition, more than 2,947 new genes were found. For functional annotation, BLASTX was used to compare the new genes to eight public databases. A total of 1,867 new genes were successfully annotated, which included 223 genes in Clusters of Orthologous Genes of proteins (COG), 843 genes in Gene Ontology (GO), 330 genes in Kyoto Encyclopedia of Genes and Genomes (KEGG), 439 genes in EuKaryotic Orthologous Groups (KOG), 655 genes in Pfam, 879 genes in SWISS-PROT, 1232 genes in EggNOG, and 1854 genes in NR.

We putatively identified expressed genes with RSEM using the reads per kb per million reads (RPKM) method (41). Genes with at least a 2-fold expression change (i.e., log2|fold change (FC)|≥1) and a false discovery rate (FDR)<0.01, as found by the DESeq R package (1.10.1), were considered differentially expressed. The GOseq R package and KOBAS software were used to implement the statistical enrichment of differentially expressed genes (DEGs) in the GO and KEGG pathways, respectively, and an adjusted Q-value < 0.05 was chosen as the significance cutoff (42).

### Expression of MMP-2 and MMP-9 in Tumors From Mice

To investigate MMP-2 and MMP-9 expression, Western blotting and immunohistochemistry (IHC) were performed on tumor tissue samples collected 12 days after tumor transplantation. Protein expression was analysed using antibodies against MMP-2 (ab37150, 1:500; Abcam, UK), MMP-9 (ab38898, 1:1000; Abcam, UK), and β-actin (1:2000; Abcam, UK). IHC was performed according to the manufacturer’s recommended protocol using rabbit anti-mouse MMP-9 (ab38898; Abcam, UK) and MMP-2 (ab37150; Abcam, UK) antibodies, and the integrated optical density (OD) intensity of IHC staining was quantified as previously reported (43).

### Therapeutic Schedules for a Selective MMP-2/9 Inhibitor

In addition, WT (n=7), STZ (n=9) and *db/db* (n=9) mice with pancreatic cancer were given an i.p. injection of the selective MMP-2/9 inhibitor SB-3CT (Selleck, USA) diluted in 10% DMSO at a dosage of 25 mg/kg daily for 20 days (44). The control groups for WT (n=9), STZ (n=9) and *db/db* (n=11) mice were administered an equal volume of vehicle (10% DMSO). Tumor growth was monitored using T2-weighted MRI. Metastatic lesions were counted in bioluminescence images. Western blotting was conducted to evaluate MMP-2 and MMP-9 expression levels.

### Therapeutic Schedules for the Combination Therapy

To investigate the potential synergistic effect of the MMP-2/9 inhibitor SB-3CT and gemcitabine combination therapy, WT (n=9), STZ (n=8), and *db/db* (n=8) mice were administered SB-3CT (25 mg/kg, i.p.) daily for 20 days. During this period, gemcitabine (25 mg/kg, i.p.) was injected every 3 days. Tumor volume and metastases were evaluated using the methods described above.

### Statistical Analysis

One-way ANOVA followed by Tukey’s test was used to examine the differences in tumor volume, the OD intensity of IHC staining (MMP-2 and MMP-9), tumor-background ratio (TBR) values, and the number of metastatic nodules in animals. A value of *p ≤* 0.05 was considered statistically significant. All analyses were performed with SPSS v.20 software (IBM SPSS, Armonk, NY, USA).

## Results

### Pancreatic Cancer Progression in Diabetic Mouse Models

**Figure 1:**
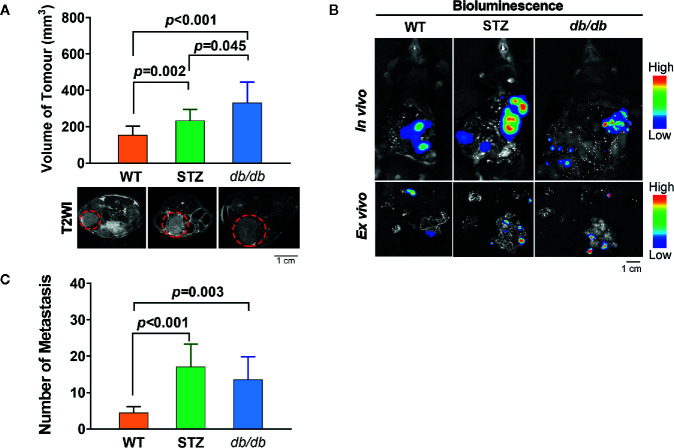
STZ-induced or genetically induced Type 2 diabetes mellitus (T2DM) promotes pancreatic cancer progression. **(A)** Tumor volume was visualized and quantified according to T2-weighted images, which were acquired by MR scans, on day 12 after tumor implantation from WT, STZ, and *db*/*db* mice. Representative T2-weighted images are presented. The red circle indicates the tumor location. **(B)** Pancreatic cancer metastasis in WT, STZ and *db*/*db* mice was evaluated with bioluminescence imaging on day 12 after tumor implantation. **(C)** Representative *in vivo* and *ex vivo* bioluminescence imaging are shown. Scale bar, 1 cm. Data are presented as the mean ± S.D.

The process of tumor progression was first investigated. Similar to previous reports, the pancreatic cancer volume in STZ (n=12, 257.70 ± 32.28 mm^3^; *t*=-3.027, *df*=24, *p*<0.05) and *db*/*db* (n=14, 312.33 ± 41.01 mm^3^; *t*=-3.593, *df*=26, *p*=0.001) mice was significantly larger than that in WT mice (n=14, 156.89 ± 13.77 mm^3^) (**Figure 1A**).

In addition, we found that the number of metastases in STZ (n=9, 32.00 ± 5.83; *t*=-3.797, *df*=15, *p*<0.05) and *db*/*db* (n=10, 21.80 ± 7.38; *t*=-1.625, *df*=16, *p*<0.05) mice was significantly higher than that in WT mice (n=8, 8.25 ± 0.86) (**Figures 1B, C**).

### Gemcitabine Treatment

**Figure 2:**
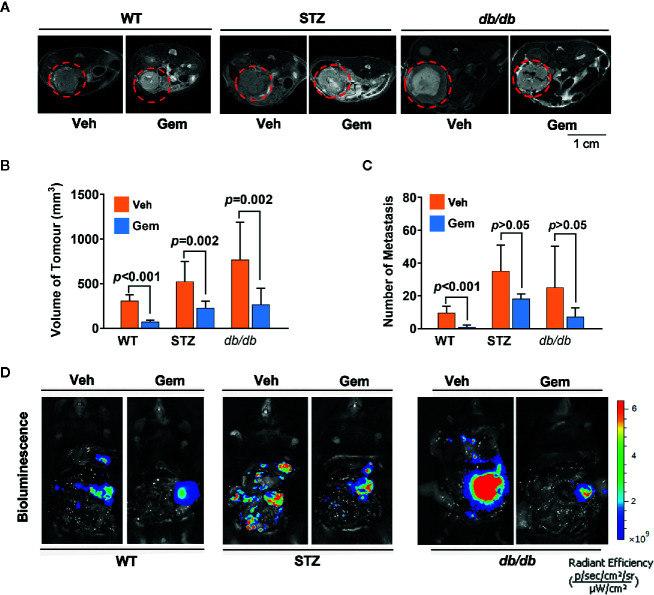
Antitumor effects in diabetic mice with pancreatic cancer after treatment with gemcitabine (Gem). **(A, B)** Gem (25 mg/kg, Sigma, i.p.) was administered every 3 days for 20 days in WT, STZ, and *db/db* mice with pancreatic cancer after tumor transplantation. Tumor volume was quantified in non-diabetic and diabetic mice with pancreatic cancer based on T2-weighted images. Representative tumor volumes based on T2-weighted images are shown from each group **(A),** and tumor volumes at 20 days after Gem treatment were calculated in each group **(B)**. The red circle indicates the tumor location. **(C, D)** Bioluminescence was analyzed in each group after Gem treatment to visualize and evaluate tumor metastasis, and the tumor metastasis number from each group at 20 days after Gem treatment was calculated **(C)**. Representative *in vivo* bioluminescence imaging is shown **(D)**. The control groups mice were administered an equal volume of vehicle (Veh). Data are shown as the mean ± S.D.

Standard chemotherapy, i.e., gemcitabine, is the first-choice chemotherapy agent for pancreatic cancer (39), which was used as a treatment in our study. The results showed that the tumor volume significantly decreased in WT, STZ and *db*/*db* mice with gemcitabine treatment (all *p*<0.05). However, gemcitabine was less effective at reducing the number of metastases (**Figure 2**).

### Transcriptome Analyses

For enhancing our knowledge, the gene expression profiling of pancreatic cancer under diabetes, was performed using RNA-seq. When comparing pancreatic cancer tissue from non-diabetic WT mice with that from obesity-associated diabetic *db*/*db* mice, a total of 64 significant differentially expressed genes (DEGs) were identified (**Table S1**). The expression of 21 and 43 genes was up- and downregulated, respectively, in pancreatic cancer tissue from *db*/*db* mice. In addition, a total of 136 DEGs were identified when comparing pancreatic cancer tissue from WT mice with that from non-obesity-associated STZ-induced diabetic mice, and the expression of 50 and 86 genes was up- and downregulated, respectively, in pancreatic cancer tissue from STZ mice (**Table S2**).

#### GO Enrichment Analyses of DEGs in *db*/*db* Mice *vs.* WT Mice

GO enrichment analyses generated two subcategories: interleukin (IL)-8 receptor activity (GO: 0004918; *p*<0.05) and enzyme binding (GO: 0019899; *p*<0.05), in which the genes with upregulated expression were enriched. Furthermore, nine subcategories were enriched in genes with downregulated expression; most of these downregulated gene-enriched subcategories, for example, MHC class II protein complex (GO: 0072558; *p*<0.05), interleukin-1α production (GO: 0032610; *p*<0.01), natural killer (NK) cell activation (GO: 0030101; *p*<0.05), NLRP1 inflammasome complex (GO: 0072558; *p*<0.01), and positive regulation of interleukin-1β secretion (GO: 0050718; *p*<0.01) are involved in the immune response (**Table 1**).

**Table 1:** GO enrichment analyses in *db*/*db* vs. WT mice.

	Ontology	GO_ID	GO_term	*p*
Downregulated genes	Molecular Function	GO:0019904	protein domain specific binding	0.015145
	Cellular Component	GO:0072558	NLRP1 inflammasome complex	4.51E-06
		GO:0042613	MHC class II protein complex	0.018398
	Biological Process	GO:0032610	interleukin-1 alpha production	8.24E-07
		GO:0070269	Pyroptosis	0.000135
		GO:0032495	response to muramyl dipeptide	0.00037
		GO:0050718	positive regulation of interleukin-1 beta secretion	0.001845
		GO:0030163	protein catabolic process	0.004336
		GO:0030101	natural killer cell activation	0.038056
Upregulated genes	Molecular Function	GO:0004918	interleukin-8 receptor activity	0.040448
		GO:0019899	enzyme binding	0.047501

The immune response is an important element in the progression of pancreatic cancer (45). Tumor cells exploit several immunosuppressive mechanisms to block the anti-tumoral response (46–48). For example, in the tumor microenvironment, the cell-surface or secreted form of IL-1α is associated with anti-tumor responses (49), and NK cells are important cytotoxic cells in the immune system and can recognize and kill cancer cells (50). While, associated genes belonging to those GO enrich subcategories were downregulated in pancreatic cancer tissue from *db*/*db* mice. It was suggested that the attenuated immune response in *db*/*db* mice with obesity-associated T2DM might be an important biological factor leading to the increased malignancy of pancreatic cancer.

#### GO Enrichment Analyses of DEGs in STZ Mice *vs.* WT Mice

GO enrichment analyses generated 16 enriched subcategories of genes with downregulated expression and 10 enriched subcategories of genes with upregulated expression, and are listed in detail in **Table 2**. Many of the enriched subcategories are involved in carcinogenesis, could be mainly divided into 3 parts, and are discussed as follows:

**Table 2:** GO enrichment analyses in STZ vs. WT mice.

	Ontology	GO_ID	GO_term	*P*
Downregulated genes	Molecular Function	GO:0004055	argininosuccinate synthase activity	0.002019
		GO:0005201	extracellular matrix structural constituent	0.042346
		GO:0042605	peptide antigen binding	0.046186
	Cellular Component	GO:0005584	collagen type I trimer	0.001452
		GO:0032398	MHC class Ib protein complex	0.021541
		GO:0005615	extracellular space	0.026812
		GO:0070852	cell body fiber	0.039994
	Biological Process	GO:0071356	cellular response to tumor necrosis factor	0.000209
		GO:0071346	cellular response to interferon-gamma	0.000209
		GO:0042832	defense response to protozoan	0.002222
		GO:0006526	arginine biosynthetic process	0.019691
		GO:0000053	argininosuccinate metabolic process	0.019691
		GO:0071230	cellular response to amino acid stimulus	0.022237
		GO:0007494	midgut development	0.035324
		GO:2000502	negative regulation of natural killer cell chemotaxis	0.039285
		GO:0060416	response to growth hormone	0.046176
Upregulated genes	Molecular Function	GO:0005172	vascular endothelial growth factor receptor binding	0.019203
	Biological Process	GO:0014842	regulation of satellite cell proliferation	0.002814
		GO:0030210	heparin biosynthetic process	0.009355
		GO:0033552	response to vitamin B3	0.014015
		GO:0007171	activation of transmembrane receptor protein tyrosine kinase activity	0.026095
		GO:0003160	endocardium morphogenesis	0.026095
		GO:0001936	regulation of endothelial cell proliferation	0.033509
		GO:0072012	glomerulus vasculature development	0.033509
		GO:0031398	positive regulation of protein ubiquitination	0.041058
		GO:0048014	Tie signaling pathway	0.041833

In addition to the enriched subcategories (e.g., MHC class Ib protein complex, GO: 0032398; cellular response to interferon-gamma, GO: 0071346) involved in the immune response associated with downregulated genes, enriched subcategories of “collagen type I trimer” (GO: 0005584) and “extracellular matrix structural constituent” (GO: 0005201) associated with the ECM were identified. A number of studies have shown that changes in the levels of ECM proteins play a major role in invasion and migration and correlate with the increased invasive potential of pancreatic cancer cells (51–54).

In addition, subcategories (e.g., vascular endothelial growth factor receptor binding, GO: 0005172; regulation of endothelial cell proliferation, GO: 0001936; and glomerulus vasculature development, GO: 0072012) involved in tumor vasculature and angiogenesis were enriched in genes with upregulated expression. Activation of the vascular endothelial growth factor (VEGF)/VEGF-RII pathway has been shown to regulate angiogenesis, and tumor vasculature and angiogenesis play an important role in tumor growth, metastasis and chemo- and radiotherapy responses (55, 56). Therefore, the abovementioned biological factors predominantly involved in the immune response, angiogenesis and ECM remodeling are likely to commonly contribute to the malignancy of pancreatic cancer in non-obese-associated T2DM, i.e., STZ-treated mice.

#### KEGG Pathway Enrichment Analyses

Consistent with the results of GO enrichment in *db*/*db* mice, pathway enrichment tests revealed that immune response-related pathway, NOD-like receptor signaling pathway (ko: 04621, **Table S3**) was enriched in genes with downregulated expression. While, in STZ mice, enriched functional categories, e.g., ECM-receptor interaction (ko: 04512), platelet activation (ko: 04611), Rap1 signaling pathway (ko: 04015), adherens junction (ko: 04520) (**Table S3**), mainly involved in carcinogenesis, which might contribute to the malignancy of pancreatic cancer in STZ-treated mice. In general, results of KEGG pathway enrichment are roughly consistent with the results of GO enrichment analyses.

#### DEGs Related to Carcinogenesis

Consistent with previous reports (23, 57, 58), in our study, pancreatic cancer malignancy (i.e., measured by tumor volume and metastasis number) was found in both obesity-associated *db*/*db* T2DM mice and non-obese-associated STZ T2DM mice. It is generally hypothesized that diabetes may influence this neoplastic process by several mechanisms associated with hyperinsulinaemia, hyperglycaemia, and chronic inflammation (8–11). Carcinogenesis is a complex process, which involves multiple steps, including initiation, promotion and progression and is mediated by a series of genes (e.g., KRAS, CDKN2A, TP53, HER2, and HIF-1α) (27–30). Our transcriptomic data identified many genes i.e., DEGs that have been reported in previous studies to be associated with carcinogenesis (**Tables S1** and **S2**), which were significantly differentially expressed in the pancreatic cancer samples from STZ or *db*/*db* mice, or both. These DEGs could be candidate genes associated with the increased malignancy of pancreatic cancer in T2DM and may serve as potential therapeutic targets, as well. Further work should be conducted to identify genes playing a critical role and to identify effective therapeutic targets from these genes.

Among the DEGs, 11 were significantly differentially expressed in pancreatic cancer samples from both STZ and *db*/*db* mice, with the same expression tendencies (**Table 3**). Consistent with the results of the GO enrichment analysis, most genes associated with carcinogenesis in STZ and *db*/*db* showed many expression differences; for example, *Csdc2* gene was upregulated in STZ mice but showed no expression differences in *db*/*db* mice (**Tables S1** and **S2**). This finding suggests that although the pancreatic cancer malignancy was increased in both obese-associated *db*/*db* T2DM and STZ mice with non-obese-associated T2DM (**Figure 1**), the increase may be caused by different genes and may also involve different mechanisms. In addition, precise and differentiated therapeutic targets may be selected according to whether pancreatic cancer patients have obesity- or non-obesity-associated T2DM.

**Table 3:** DEGs in both *db*/*db* ad STZ.

ID	Gene name	WT *vs.* STZ	WT *vs.* *db*/*db*	NR annotation
ENSMUSG00000061577	Gpr114	Down	Down	probable G-protein coupled receptor 114 isoform 1 precursor [Mus musculus]
ENSMUSG00000021200	Asb2	Down	Down	ankyrin repeat and SOCS box protein 2 [Mus musculus]
ENSMUSG00000015852	Fcrls	Up	Up	Fc receptor-like S, scavenger receptor precursor [Mus musculus]
ENSMUSG00000017737	Mmp9	Up	Up	matrix metalloproteinase-9 precursor [Mus musculus]
ENSMUSG00000058126	Tpm3-rs7	Down	Down	tropomyosin alpha-3 chain isoform 3 [Mus musculus]
ENSMUSG00000091679	Vmn2r96	Down	Down	vomeronasal 2, receptor 96 [Mus musculus]
ENSMUSG00000031740	Mmp2	Up	Up	72 kDa type IV collagenase precursor [Mus musculus]
ENSMUSG00000022501	Prm1	Down	Down	sperm protamine P1 [Mus musculus]
ENSMUSG00000055546	Timd4	Down	Down	T-cell immunoglobulin and mucin domain-containing protein 4 precursor [Mus musculus]
ENSMUSG00000023827	Agpat4	Up	Up	1-acyl-sn-glycerol-3-phosphate acyltransferase delta [Mus musculus]
ENSMUSG00000030607	Acan	Up	Up	aggrecan core protein precursor [Mus musculus]

In addition to selecting differentially expressed candidate genes for therapeutic targets, we also attempted to explore the common targets according to the 11 genes differentially expressed in pancreatic cancer from both *db*/*db* and STZ mice for both obese- and non-obese-associated T2DM. For example, the expression of MMP-2 and MMP-9 in the abovementioned 11 DEGs was found to be upregulated in the pancreatic cancer samples from both STZ and *db*/*db* mice and were further researched in subsequent analyses (see *Therapeutic Targets of MMP-2 and MMP-9*). Theoretically, common therapeutic targets have a wide range of applications for pancreatic cancer in both non-obese- and obese-associated *db*/*db* T2DM patients.

### Expression of MMP-2 and MMP-9 in Tumors From Mice

**Figure 3:**
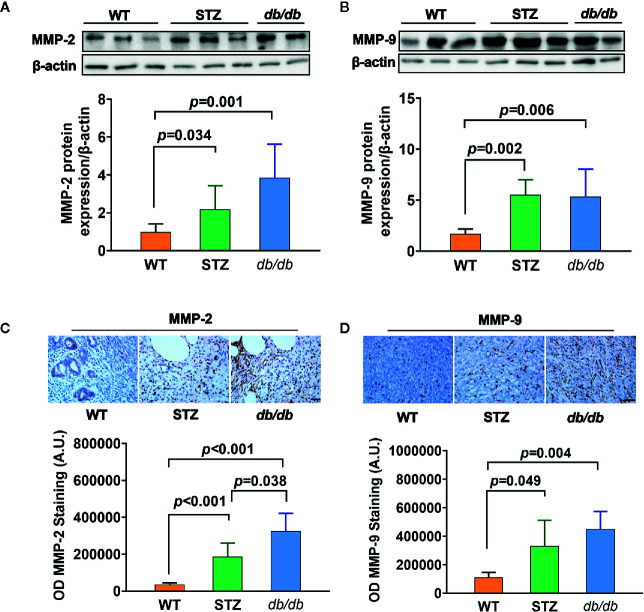
MMP-2 and MMP-9 expression levels were higher in pancreatic cancer with Type 2 diabetes mellitus (T2DM) than in pancreatic cancer without T2DM. **(A, B)** Western blot analysis of MMP-2 and MMP-9 was conducted in tumors in the WT, STZ, and *db*/*db* groups on day 12 after tumor implantation. MMP-2 **(A)** and MMP-9 **(B)** levels were quantified by Image-Pro Plus and normalized to β-actin. **(C, D)** Expression levels of MMP-2 **(C)** and MMP-9 **(D)** in pancreatic cancer tissues from WT, STZ and *db*/*db* mice were also evaluated with immunohistochemistry (IHC) on day 12 after tumor implantation. Data are representative of MMP-2 and MMP-9 staining in pancreatic cancer from the different mouse models, and the quantified integrated optical density (OD) value was calculated. Scale bar, 100 µm. Data are presented as the mean ± S.D.

Transcriptomic data further showed, as previously described (results of qPCR) (58), that the expression of the genes encoding MMP-2 and MMP-9 was upregulated in pancreatic cancer tissues from both STZ and *db*/*db* mice; this result was also confirmed by Western blotting (**Figures 3A, B**) and IHC analyses (**Figures 3C, D**, all *p*<0.05).

### Therapeutic Targets of MMP-2 and MMP-9

**Figure 4:**
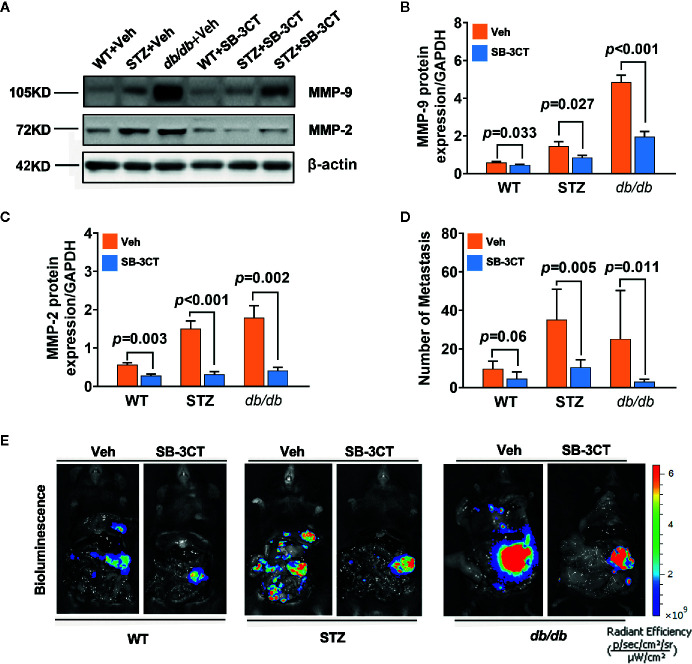
Antitumor effects in diabetic mice with pancreatic cancer after being treated with an MMP-2/9 inhibitor. **(A, C)** Western blot analysis of MMP-2 and MMP-9 in pancreatic cancer in WT, STZ, and *db/db* mice after treatment with an MMP-2/9 inhibitor (SB-3CT, 25 mg kg-1 day-1) or vehicle (Veh) for 20 days. MMP-9 **(B)** and MMP-2 **(C)** levels were quantified by Image-Pro Plus and normalized to β-actin. **(D, E)** Bioluminescence was analysed in each group to visualize and evaluate tumor metastasis at 20 days after the generation of the model. The number of tumor metastases was calculated **(D),** and representative *in vivo* bioluminescence imaging is presented **(E)**. Data are shown as the mean ± S.D.

When STZ and *db*/*db* mice bearing pancreatic cancer were treated with a selective inhibitor of MMP-2 and MMP-9, SB-3CT, for 20 days in this study, the expressions of MMP-2 and MMP-9 significantly decreased (all p<0.05) (**Figures 4A–C**). In addition to tumor volume changes (58), the number of metastases significantly decreased, as well (**Figures 4D, E**).

### Comparison of the Therapeutic Effect of an MMP-2/9 Inhibitor and Gemcitabine

**Figure 5:**
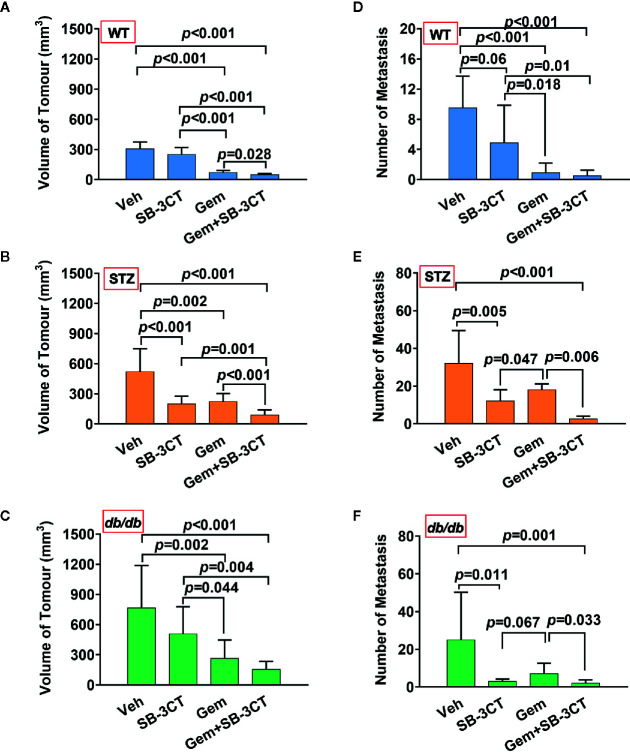
The antitumor effects of vehicle (Veh), an MMP-2/9 inhibitor (SB-3CT), gemcitabine (Gem) or a combination of gemcitabine and an MMP-2/9 inhibitor were analysed in WT, STZ, and *db/db* mice with pancreatic cancer. Pancreatic tumor volumes were calculated based on T2-weighted images after four different treatments for 20 days and were compared in the WT **(A)**, STZ **(B)**, and *db/db* **(C)** groups. Metastasis numbers were quantified according to bioluminescence imaging after four different treatments for 20 days and were analysed in the WT **(D)**, STZ **(E)**, and *db/db* **(F)** groups. Data are shown as the mean ± S.D.

Additionally, to better understand and compare the therapeutic effect of targeting MMP-2 and MMP-9 with SB-3CT, a standard chemotherapy for pancreatic cancer, i.e., gemcitabine, was used. The results showed that compared with inhibitor treatment, gemcitabine treatment had a greater effect on tumor volume (**Figures 5A, B**); however, gemcitabine was less effective at reducing the number of metastases (**Figures 5C, D**).

### Optimizing the Therapeutic Strategy by Combining an MMP-2/9 Inhibitor With Gemcitabine

Unlike primary tumors, metastasis is largely incurable and contributes to more than 90% of cancer-associated tumors (59). Considering the abovementioned shortcomings of using chemotherapy or the inhibitor alone, a therapeutic strategy combining these two treatments was implemented. The results showed that the combination therapy was significantly more effective than either gemcitabine or MMP-2/9 inhibitor treatment alone for both non-obesity- and obesity-associated diabetic pancreatic cancer, resulting in the smallest tumor volume and lowest number of metastases (**Figure 5**, all *p*<0.05).

## Discussion

An increasing amount of evidence has shown that diabetes is an important risk factor for many cancers (2–5), including pancreatic cancer, as higher morbidity and lower survival has been observed in pancreatic cancer patients with T2DM (17–20). This study provides supporting evidence for the promotion of pancreatic cancer progression by diabetes, with accelerated tumor growth and a higher degree of metastasis in diabetic individuals. There are a number of shared risk factors in both diabetes and cancers, such as obesity and hyperinsulinaemia, that have been considered as possible explanations for this link (6–11). Our study also provides supporting evidence that obesity is a potential links between diabetes and pancreatic cancer, as in our study, obesity-associated *db/db* mice presented more aggressive pancreatic tumor progression than non-obesity-associated STZ mice.

Currently, a growing amount of observational evidence suggests that therapies for diabetes can decrease the incidence and mortality of cancer (e.g., breast cancer) in patients with diabetes (12). However, recent studies have shown that the risk of pancreatic cancer might not be suppressed by anti-diabetes therapy (14). In addition, the standard chemotherapy for pancreatic cancer, i.e., gemcitabine, was used as well, but the efficacy was not satisfactory. As shown in many clinical studies (22, 23), diabetic individuals who undergo adjuvant chemotherapy for pancreatic cancer present with larger tumors (also see our results) and have a smaller reduction in the metastasis number. Thus, the unsatisfactory efficacy of current therapeutic strategies inspired us to explore other treatment strategies or therapies.

Carcinogenesis is a complex process that involves multiple steps, including initiation, promotion and progression, and is mediated by a number of genes and proteins (27–30). Thus, when diabetes promoted the progression of cancer, transcriptomic analysis results showed that there were 11 genes that were differentially expressed in pancreatic cancer tissues from both the STZ and *db*/*db* mice that might participate in and play a role in promoting cancer progression. Among these genes, our study further proved that the expression of both MMP-2 and MMP-9 was abnormally upregulated in pancreatic cancer tissue under diabetic conditions. MMP-2 and MMP-9 belong to a family of MMPs known to be gelatinases that promote the degradation of type IV collagen in the basement membrane during cancer cell invasion and metastasis (60–63). In our study, when mice with pancreatic cancer were treated with a selective inhibitor of MMP-2 and MMP-9, SB-3CT, tumor volume (58) and the number of metastases significantly decreased (**Figures 3D, E**).

Overall, our study suggested that targeting MMP 2 and 9 is an alternative strategy that could achieve satisfactory therapeutic efficacy, as the MMP-2/9 inhibitor SB-3CT reduced malignancy, especially in terms of metastasis. In addition, in terms of tumor size, gemcitabine was more effective than the inhibitor treatment. To combine the abovementioned advantages of using chemotherapy or inhibitors alone, an optimized therapeutic strategy was further proposed and implemented that combined an MMP-2/9 inhibitor with gemcitabine. Optimizing the therapeutic strategy significantly enhanced the antitumor effects on pancreatic cancer with concomitant diabetes and provided a basis for clinical applications.

However, the mechanism of abnormally upregulated MMP-2 and MMP-9 expression in pancreatic cancer from diabetic STZ and *db/db* mice was unclear. The following two possible explanations were proposed. One explanation is that diabetes induces the promotion of pancreatic cancer progression, then cancer cell frequency synthesis of MMP for adapting rapid invasion and metastasis. Alternatively, upregulated MMP-2 and MMP-9 expression might be caused by abnormal metabolic stimuli resulting from the presence of diabetes, which is known to induce the dysfunction of several intracellular signal transduction cascades; for example, protein kinase activity and the accumulation of advanced glycation end products might induce the generation of MMP overproduction under diabetic conditions (64–68). Generally, either cancer itself, diabetes, or both induced abnormal MMP-2 and MMP-9 overproduction, which should be further studied to address these unanswered questions.

## Conclusions

Consistently, pancreatic cancer in the current study showed increased proliferation and metastatic potential in both obesity-associated and non-obesity-associated T2DM. To widen our knowledge of diabetes as a risk factor for the development of pancreatic tumors, gene expression profiling was performed using RNA-seq. The transcriptomic analysis results showed that there were 136 and 64 DEGs in STZ and *db*/*db* mice, respectively. Among these DEGs, many are associated with carcinogenesis. This result provides useful information about potential molecular factors contributing to the malignancy of pancreatic cancer. Our results also suggest that the carcinogenesis-related genes contribute to the malignancy of pancreatic cancer are different in obesity-associated and non-obesity-associated T2DM. In addition, we found MMP-2 and MMP-9 as common therapeutic targets among 11 genes differentially expressed in both the STZ and *db*/*db* mice. These genes could be targeted to suppress the malignant progression of pancreatic cancer in both obesity-associated and non-obesity-associated T2DM. Thus, in addition to testing current therapeutic strategies against diabetes, our study offers a potential therapeutic strategy in which MMPs serve as a therapeutic target in combination with chemotherapy for diabetic pancreatic cancer patients, thus providing a basis for further study and a theoretical basis for clinical applications. In addition, the results also suggested that the choice of medication should be based on the individual’s conditions; for example, the inhibitor had a lower therapeutic effect on pancreatic cancer alone; while the combination therapy may represent a better option for pancreatic cancer when diabetes is also present.

## Data Availability Statement

The datasets presented in this study can be found in online repositories. The names of the repository/repositories and accession number(s) can be found below: NCBI Sequence Read Archive database (BioProject ID: PRJNA661998).

## Ethics Statement

The animal study was reviewed and approved by Institutional Animal Care and Use Committee of the Medical School of Southeast University.

## Author Contributions

TX conceived and performed the experiments. XX and P-CL analyzed the data. Both TX and P-CL wrote the paper. SJ and HM revised the manuscript. All authors contributed to the article and approved the submitted version.

## Funding

This work was supported by grants from the National Nature Science Foundation of China (NSFC, No. 81525014, No. 82001888 and No. 81830053) and the Key Research and Development Program of Jiangsu Province (BE2016782).

## Conflict of Interest

The authors declare that the research was conducted in the absence of any commercial or financial relationships that could be construed as a potential conflict of interest.
